# Modeling the Resistance Evolution to Insecticides Driven by Lepidopteran Species Competition in Cotton, Soybean, and Corn Crops

**DOI:** 10.3390/biology11091354

**Published:** 2022-09-15

**Authors:** José B. Malaquias, Cláudia P. Ferreira, Francisco de S. Ramalho, Wesley A. C. Godoy, Jéssica K. S. Pachú, Celso Omoto, Dyrson de O. A. Neto, Fernando E. O. Padovez, Luciana Barboza Silva

**Affiliations:** 1 Department of Biostatistics, Institute of Biosciences–IBB, São Paulo State University (UNESP), Botucatu 18618-693, Brazil; 2 Biological Control Unit, Embrapa Algodão, Av. Osvaldo Cruz, 1143 Campina Grande, Paraíba 58107-720, Brazil; 3 Department of Entomology and Acarology, Luiz de Queiroz College of Agriculture (ESALQ), University of São Paulo (USP), Av. Pádua Dias 11, Piracicaba 13418-900, Brazil; 4 Campus Professora Cinobelina Elvas, Federal University of Piauí, Bom Jesus, Piauí 64900-000, Brazil

**Keywords:** evolutionary dynamics, pesticides, ecological interactions, competition, refuge

## Abstract

**Simple Summary:**

Lepidopteran species commonly interact in the same niches in multiple crops. Interspecific competition has been neglected as a pressure selection agent in insecticide resistance studies. Our results showed that competition may act as an agent to speed up the evolution of diamide resistance in *Spodoptera frugiperda* and *Helicoverpa armigera*.

**Abstract:**

Intra- and interspecific competition is considered a fundamental phenomenon in ecology. It acts as one of the most powerful selective forces that drives ecological diversity, the spatiotemporal distribution of organisms, fitness, and evolutionary aspects. *Spodoptera frugiperda* and *Helicoverpa armigera* are devastating pests and can co-occur in systems consisting of multiple agricultural crops and compete for food resources. Insecticide resistance in populations of these species has been a major threat to the sustainability of agroecosystems. No study to date has shown the effect of intra- and interspecific competition as a selective pressure agent on the evolution of insecticide resistance in lepidopteran pests in an experimental and theoretical way. Our study developed a parameterized computational model with experimental results for *S. frugiperda* and *H. armigera* competition. We simulated the behavior of heterozygous individuals with a competition capacity 100% equal to homozygous individuals resistant (100 RR) or susceptible to insecticides (00 RR), and intermediate between them (50 RR). Competition involving strains of these insect species can accelerate the evolution of their resistance to insecticides in agricultural crops. We found that competitive processes can result in a high probability of competitive exclusion for individuals with the susceptibility allele of these lepidopteran species. The results of this study are of paramount importance for understanding the impact of ecological factor competition on the evolution of insecticide resistance in lepidopteran pests, which until now has been neglected in these types of evolutionary dynamics studies.

## 1. Introduction

Generalist herbivorous insects commonly interact by sharing the same niches [1,2]; however, in many cases, competitors do not share the same niche. *Spodoptera frugiperda* (J.E. Smith) (Lep.: Noctuidae) and *Helicoverpa armigera* (Hübner) (Lepidoptera: Noctuidae) are generalist herbivores that have the same niche in tropical agroecosystems, thus attacking the vegetative and reproductive structures of their host plants, such as cotton (*Gossypium hirsutum* L.), corn (*Zea mays* L.), and soybean (*Glycine max* (L.) Merr.) [3,4,5]. These crops are planted in adjacent agricultural mosaics or in succession, which serve as a green bridge and facilitate the movement and colonization of *S. frugiperda* and *H. armigera* between crops, promoting a scenario of high selection pressure imposed by the application of insecticides.

In tropical agroecosystems, the efficacy of insecticides and genetically modified crops is threatened by the rapid evolution of resistance in these pest insects. One major concern is that the evolution of resistance of *S. frugiperda* and *H. armigera* usually occurs in a few generations of the insect, including resistance to diamides [6,7], which until recently has been highly efficient against these larvae, reinforcing the search for knowledge regarding the bioecology and interactions involving host–insect and insect–insect relationships.

To prevent or significantly delay insect resistance to Bt crops, such as cotton, corn, or soybean, refuge areas should be planted with non-Bt plants and cover a significant percentage of the acreage in the field [8,9,10,11]. Malaquias et al. [8] analyzed how the adoption of insecticide and the competition between the lepidopteran species *S. frugiperda* and *H. armigera* could impact the production of susceptible individuals in refuge areas of Bt cotton. They warned that ecological dominance may diverge due to resistance alleles because insecticide resistance in *S. frugiperda* could impact the production of susceptible *H. armigera* individuals on a large scale in refuge areas. In their study, the competition between the resistant and susceptible strains was not analyzed. Such evidence encourages further experimental studies approaching the competition of these insects involving *S. frugiperda* and *H. armigera* strains considering resistance, heterozygosity, and susceptibility to insecticides.

Knowledge regarding the competitive potential of lepidopteran species allows more accurate inferences about the consequences of competitive dynamics for the evolution of insect resistance to insecticides used in refuge areas of transgenic plants. However, measuring the existence and effects of intra- and interspecific competition in a phenotypic context on the evolutionary dynamics of competing species is a considerable challenge. For this purpose, the combined use of experimentation techniques and computational modeling is necessary [12].

A model is a simplified representation of the essential elements of a behavioral system that can make testable predictions. Game theory provides a mathematical framework for examining the evolution of behavioral strategies that can be employed during competition [13]. A game model can be defined as a competitive activity involving skill, chance, or endurance on the part of two or more players who make strategic decisions [8]. Mathematical games have strict rules and specify what is allowed and what is not. Similarly, cellular automata models are based on the specification of rules for modifying the state of the cell and its neighbors. The state of the cell can be represented from the ecological point of view by the absence and presence of the individual or the species [14]. Thus, the behavioral decision-making of competing or not competing can be studied using models based on cellular automata and with rules inspired by game theory [8].

Given the context presented, the present study sought to understand the role of intra- and interspecific competition involving *S. frugiperda* and *H. armigera* strains in the evolution of their resistance to flubendiamide in cotton, corn, and soybean crops. Considering the fitness cost of the resistant strain in the absence of selective pressure, and the fact that the primary host of *S. frugiperda* is corn, and the fitness of *H. armigera* has proven to be superior in cotton and soybean compared to corn, we hypothesized that competitive performance would be given according to the strain of the insects and the host plant (i). Even in the absence of selective pressure, phenotypic interactions would be sufficient to influence the resistance evolution in these lepidopteran pests to insecticides in these crops (ii). Assuming that cannibalism has a great impact on insect fitness, we also believe that cannibalism can control the speed of resistance evolution (iii). As we did not collect data concerning the competitive behavior of the heterozygotes, we needed to simulate it. Thus, we also explored possible conditions by which heterozygotes might behave in relation to resistant and susceptible strains.

## 2. Materials and Methods

### 2.1. Bioassays

Two experiments were performed independently under greenhouse conditions for each host plant species (cotton, corn, or soybean). We used resistant (RR) and susceptible (SS) strains of *S. frugiperda* and *H. armigera* to flubendiamide selected by [6,7]. Both populations have been maintained at the Arthropod Resistance Laboratory, Department of Entomology and Acarology, Luiz de Queiroz College of Agriculture (Escola Superior de Agricultura “Luiz de Queiroz”-ESALQ/USP), Piracicaba, São Paulo state, Brazil. These experiments were conducted in a climate-controlled greenhouse at 25 ± 1 °C, with a relative humidity of 65 ± 10% and a 12-h photophase. To avoid the effect of abiotic factor variation all experiments were conducted at once.

In the first experiment, intra- and interspecific competition involving susceptible (SS) and resistant (RR) strains of *S. frugiperda* (Sf) and *H. armigera* (Ha) was evaluated, which consisted of the following treatments: Ha-SS vs. Sf-SS (Treatment 1); Ha-SS vs. Sf-RR (Treatment 2); Ha-RR vs. Sf-SS (Treatment 3); Ha-RR vs. Sf-RR (Treatment 4).

In the second experiment, only the intraspecific interactions were evaluated, with the following combinations for *H. armigera* or *S. frugiperda*: Ha-SS vs. Ha-SS (Treatment 1); Ha-SS vs. Ha-RR (Treatment 2); Ha-RR vs. Ha-RR (Treatment 3); Sf-SS vs. Sf-SS (Treatment 4); Sf-SS vs. Sf-RR (Treatment 5); and Sf-RR vs. Sf-RR (Treatment 6).

In both experiments, we adopted a density of 10 larvae with 5 larvae of each species/strain per cage of 3rd instar. The larvae were confined for five days into PVC arenas (30 cm high, 27 cm diameter) covered with an organza-type fabric containing five cotton, corn, or soybean plants individualized in each replication. We used a density of 10 larvae per cage in our experiments because Malaquias et al. [8] showed that this density is enough to promote consistent competitive dynamics.

A randomized block design with four replicates was used. Each experimental unit was represented by a cage. None of the cultivars used in the experiments expressed a Bt gene because our objective was to simulate the competitive performance of insect strains in Bt crop refuge areas (non-Bt plants). The soybean cultivar used was ‘BMX Power RR’, and the cotton and corn cultivars were ‘FM 993’ and ‘Status TG’ (Syngenta^®®^, Basel, Switzerland), respectively. Competitive performance was assessed by measuring the survival of individuals of each species.

The cotton, corn, and soybean plants were infested in the V4 vegetative stage. Artificial infestation occurred at the apex of the plants and was conducted with the aid of a brush. The number of surviving specimens was quantified daily for five days. The survival curves were estimated by the Kaplan-Meier method and compared by the log rank test. We produced survival graphs with the survminer [15] and survival [16] packages in R software [17].

### 2.2. Computational Model

A probabilistic, two-dimensional model of cellular automata with dimensions of 100 × 100 cells, parallel update rules, periodic boundary conditions, and a Moore neighborhood with a radius of 1 was programmed in R [17] and parameterized based on the data obtained in the experiments, considering only the intraspecific interactions (case 1), i.e., only interactions between individuals of the same species, and intraspecific + interspecific interactions (case 2), occurring in the latter case with simultaneous interactions between individuals of the same and different species.

**Figure 1:**
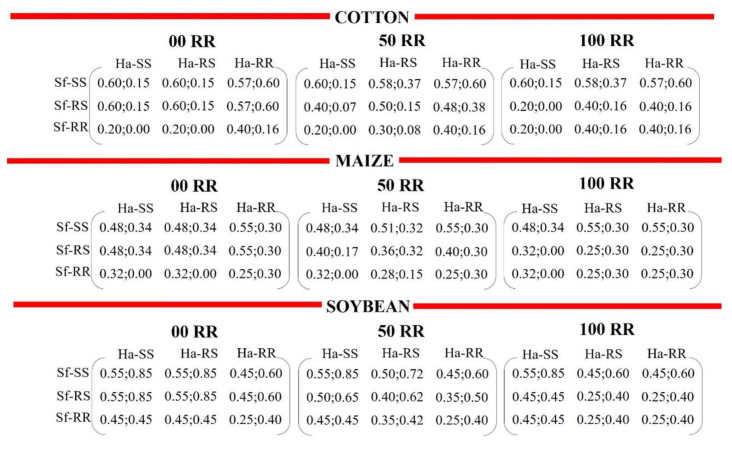
Matrices containing the survival probabilities of susceptible (SS), heterozygous (RS), and resistant (RR) strains of *Helicoverpa armigera* (Ha) and *Spodoptera frugiperda* (Sf) in cotton, corn, and soybean plants. The matrices represent the payoffs given the interspecific interactions. Data before the semicolon in each cell correspond to the survival of *S. frugiperda*, while the data after the semicolon are from *H. armigera*: 100 RR, competition behavior of heterozygous strain 100% equal to the behavior of the resistant homozygous strain; 50 RR, intermediate behavior of heterozygous strain between susceptible and resistant homozygotes; 00 RR, homozygous behavior of heterozygous strain 100% equal to the susceptible homozygote.

The rules of occupation and vacancy of the sites inspired by game theory were identical to those used by Malaquias et al. [8]. Each site (cell) of the automata independently represented a cotton, corn, or soybean plant. Each time step, t, corresponded to one generation of *H. armigera* and/or *S. frugiperda*. In the case of encounters between strains/species, the probability of survival was given by intra- and interspecific interactions (Figure 1).

In the model, we used a structured refuge with non-Bt plants; therefore, the refuge did not have exposure to any toxin. As only the interactions involving susceptible homozygous and resistant homozygous individuals were quantified in the assays, the behavior of the heterozygote was simulated with three types of conditions: 100% competition behavior equal to the behavior of the resistant homozygote (100 RR); intermediate behavior between susceptible and resistant homozygotes (50 RR); and 100% homozygous behavior equal to the susceptible homozygote (00 RR; Figure 1). The competition behavior used in the simulations was translated as the competitive performance of insects, i.e., survival given the intraspecific (only competition between individuals of the same species) and intraspecific (competition between individuals of different species) interactions.

Heterozygous individuals with competition behavior 100% equal to the homozygotes showed the same survival rates as the homozygous individuals resistant (100 RR) or susceptible to insecticides (00 RR), according to the interactions between the species, as shown in Figure 1. For example, in *H. armigera*, the survival rate in heterozygous of *H. armigera* (Ha-RS) was equal to the survival rate of susceptible homozygous of *H. armigera* (Ha-SS) at 00 RR. The survival rate of heterozygous of *H. armigera* (Ha-RS) was equal to the survival rate of resistant homozygous of *H. armigera* (Ha-RR) at 100 RR, and the same method was applied to *S. frugiperda*. For those with intermediate behavior, the median survival rates were calculated based on homozygotes survival rate (Figure 1). The median formula used was {(n + 1) ÷ 2}th, where “n” is the number of samples in the set and “th” is the (n)th number of samples.

A random selection of a random sample ranging from 0.00001 to 1 was conducted in each cell of the automata to verify compliance with the probability of an encounter between the strains/species. The probability of encountering the strains of these two species was given according to the occupancy rate of the individuals of each strain/species in the grid of 3 × 3 cells [8].

An empty site was occupied with probability W due to the oviposition of insects in the Moore neighborhood of radius 1. The probability W was given by the relative fitness. Therefore, the colonization ability of the new sites considered in the model was based on relative fitness, in which the number of eggs and the hatching rate of immature individuals were taken as references, according to the biological data of *H. armigera* and *S. frugiperda* collected in cotton, corn, and soybean [3,4,5,18]. As fitness cost is common in insecticide-resistance, we considered that the resistant individuals had 25% fitness cost in the reproduction capacity in relation to susceptible individuals.

**Figure 2:**
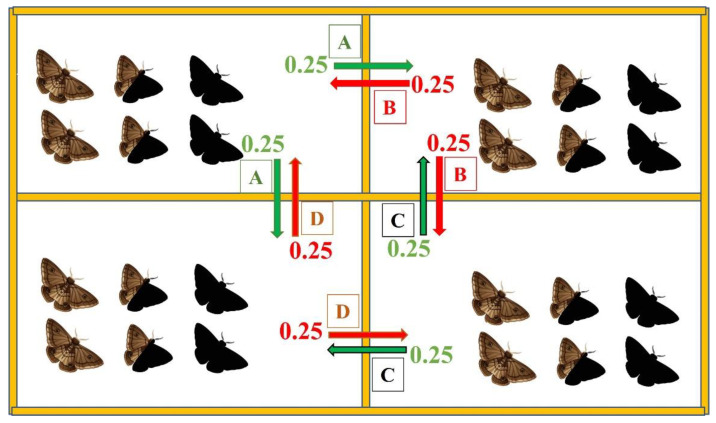
Representation of the movement of *Helicoverpa armigera* or *Spodoptera frugiperda* adults by generation in the cellular automata. The permanence and migration rates for each neighboring quadrant of adults in each quadrant were 0.50 and 0.25, respectively. Moths with black wings represent an insecticide-resistant strain; moths with brown and black wings represent a heterozygous strain; moths with brown wings represent an insecticide-susceptible strain. Green arrows indicate emigration, while red arrows indicate immigration.

Reproductive success was obtained by multiplying the number of insects of each species in the Moore neighborhood by their respective reproductive capacity (Ro). The cell network was divided into quadrants A, B, C, and D. To estimate the relative fitness, the reproductive success of each insect species that reached adulthood in each quadrant was calculated. Fifty percent of the adult individuals of each species remained in the same quadrant, while the remaining 50% of the individuals were moved to neighboring quadrants, with 25% to each of the neighboring quadrants (Figure 2). Insect mating occurred among the individuals who remained in the quadrants, along with those individuals who immigrated to each of them (quadrants A, B, C, or D).

The population genetic model used in this study is biallelic and generational, with simulations based on the relative frequency of alleles, where the allele that confers insecticide susceptibility is represented by the letter “S.” The other allele confers resistance to the mortality factor, in this case, insecticides (R). To allow encounters between all genotypes, an initial resistance allele frequency of 0.1 was used. We presumed that the population was in Hardy–Weinberg equilibrium at the beginning of the simulation and that the inheritance of resistance was autosomal, incompletely recessive, and monogenic [6,7]. In all cases, random mating was presumed among all adults of the same species in each generation (time step). The model was run 50 times with 100-time steps.

## 3. Results

### 3.1. Bioassays

#### 3.1.1. Cotton Plants

**Figure 3:**
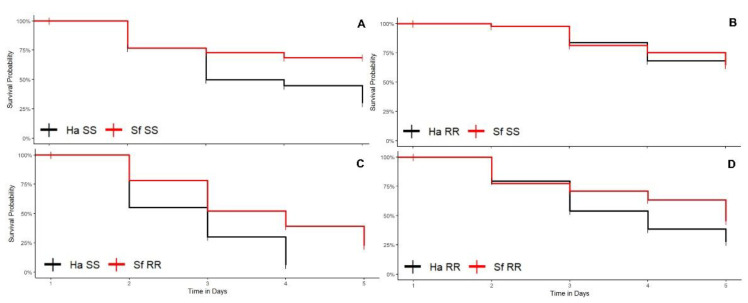
Larval survival curves of strains susceptible (SS) and resistant (RR) to insecticides of *Helicoverpa armigera* (Ha) and *Spodoptera frugiperda* (Sf) maintained in competition in cotton plants. (**A**) Ha-SS versus Sf-SS interaction; (**B**) Ha-RR versus Sf-SS interaction; (**C**) Ha-SS interaction with Sf-RR and (**D**) Ha-RR interaction with Sf-RR.

In the combination involving susceptible *H. armigera* vs. susceptible *S. frugiperda* (Ha-SS vs. Sf-SS interaction), the survival rate (ρs) of *S. frugiperda* (ρs = 0.60) was significantly higher than *H. armigera* (ρs = 0.15) (log rank test < 0.05; Figure 3A). There was no difference between the survival curves (log rank test > 0.05; Figure 3D) of the resistant strain of *H. armigera* and the susceptible strain of *S. frugiperda* (Ha-RR vs. Sf-SS interaction) (Figure 3B) and the resistant strain of *H. armigera* and the resistant strain of *S. frugiperda* (Ha-RR vs. Sf-RR interaction).

The comparisons between interspecific combinations within each species in cotton plants revealed that the competitive performance of *S. frugiperda* was higher in the interaction of the resistant strain of *H. armigera* and the susceptible strain of *S. frugiperda* (Ha-RR vs. Sf-SS interaction) (ρs = 0.57) than in the interaction between the susceptible strain of *H. armigera* and the resistant strain of *S. frugiperda* (Ha-SS vs. Sf-RR interaction) (ρs = 0.20) (log rank test < 0.05). Pairwise comparisons showed that the lowest competitive performance of *H. armigera* in cotton occurred when the susceptible larvae competed with resistant *S. frugiperda* larvae because there was no survival of susceptible *H. armigera* larvae (ρs = 0.00) in competition with resistant *S. frugiperda* (Ha-SS vs. Sf-RR interaction) (ρs = 0.20) (log rank test < 0.05) (Figure 3C).

In cotton, survival did not differ between the phenotypic combinations evaluated within the intraspecific interactions of *S. frugiperda* (log rank test > 0.05). For *H. armigera*, the survival rate between resistant and susceptible strains (Ha-RR vs. Ha-SS interaction) (ρs = 0.175) was significantly lower (log rank test < 0.05) than between resistant strains (Ha-RR vs. Ha-RR interaction) (ρs = 0.40).

The survival rate of *S. frugiperda* in the interaction between resistant *S. frugiperda* and susceptible *S. frugiperda* (Sf-RR vs. Sf-SS interaction) (ρs = 0.175) in cotton plants was significantly lower than in *H. armigera* in the interactions between susceptible individuals (Ha-SS vs. Ha-SS interaction) (ρs = 0.375) and resistant individuals (Ha-RR vs. Ha-RR interaction) (ρs = 0.400), indicating a higher rate of mortality occurred by cannibalism (personal observation) when there is competition between susceptible and resistant *S. frugiperda* larvae (log rank test < 0.05) compared to *H. armigera*.

#### 3.1.2. Corn Plants

**Figure 4:**
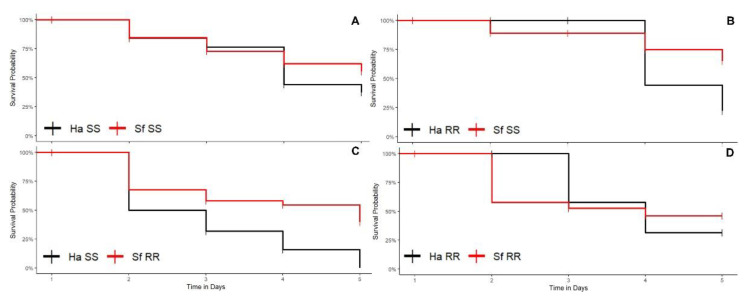
Larval survival curves of insecticide-susceptible (SS) and insecticide-resistant (RR) strains of *Helicoverpa armigera* (Ha) and *Spodoptera frugiperda* (Sf) maintained in competition in corn plants. (**A**) Ha-SS versus Sf-SS interaction; (**B**) Ha-RR versus Sf-SS interaction; (**C**) Ha-SS interaction with Sf-RR; and (**D**) Ha-RR interaction with Sf-RR.

In the interspecific comparisons in corn plants, no susceptible *H. armigera* larvae (ρs = 0.00) survived when competing with resistant *S. frugiperda* (ρs = 0.25) (log rank test < 0.05) (Ha-SS vs. Sf-RR interaction) (Figure 4C). The comparative analysis of the survival curves also showed that the competitive performance of the resistant strain of *H. armigera* (ρs = 0.30) was significantly lower than the susceptible strain of *S. frugiperda* (ρs = 0.55) (log rank test < 0.05) (Figure 4B) (Ha-RR vs. Sf-SS interaction). In contrast, there was no significant difference between the survival curves of the species in the interactions between susceptible strains of *H. armigera* and *S. frugiperda* (Ha-SS vs. Sf-SS interaction) (Figure 4A) and resistant strain of *H. armigera* and resistant strain of *S. frugiperda* (Ha-RR vs. Sf-RR interaction) (Figure 4D).

The comparisons between interspecific combinations within each species in corn plants revealed that there was no significant difference in relation to the competitive performance of *S. frugiperda* between the analyzed combinations (log rank test > 0.05). Similar to cotton, the lowest survival rate of *H. armigera* in corn plants occurred when in competition between susceptible and resistant *S. frugiperda* (log rank test < 0.05).

In corn, there was no difference between the survival rate between the phenotypic combinations evaluated within the intraspecific interactions of *S. frugiperda* (log rank test > 0.05). In *H. armigera*, survival in the interactions between resistant and susceptible strains (Ha-RR vs. Ha-SS interaction) (ρs = 0.125) was significantly lower (log rank test < 0.05) in relation to the interactions involving only susceptible strains (Ha-SS vs. Ha-SS interaction) (ρs = 0.25) and only resistant strains (Ha-RR vs. Ha-RR interaction) (ρs = 0.38). Therefore, the highest rate of cannibalism was found when the resistant strain of *H. armigera* competed with the susceptible strain of *H. armigera* (Ha-RR vs. Ha-SS interaction).

The survival rate of *H. armigera* in the interaction between resistant and susceptible individuals (Ha-RR vs. Ha-SS interaction) was significantly lower than in all interactions recorded in *S. frugiperda* in corn plants, i.e., between susceptible individuals (Sf-SS vs. Sf-SS interaction) (ρs = 0.325), between resistant and susceptible individuals (Sf-RR vs. Sf-SS interaction) (ρs = 0.500), and between resistant individuals (Sf-RR vs. Sf-RR interaction) (ρs = 0.475). This indicates a higher rate of cannibalism when there is competition between susceptible and resistant *H. armigera* larvae (log rank test < 0.05) compared to *S. frugiperda* larvae in corn plants.

#### 3.1.3. Soybean Plants

**Figure 5:**
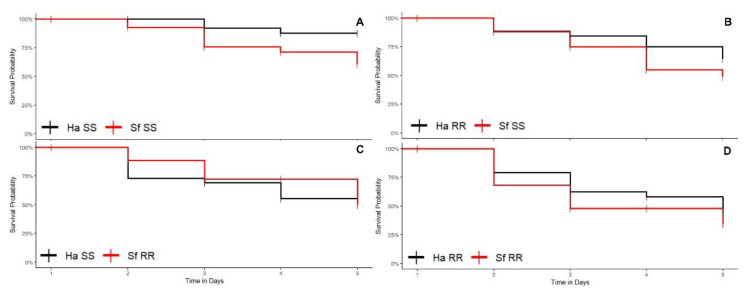
Larval survival curves of strains susceptible (SS) and resistant (RR) to insecticides of *Helicoverpa armigera* (Ha) and *Spodoptera frugiperda* (Sf) maintained in competition in soybean plants. (**A**) Ha-SS versus Sf-SS interaction; (**B**) Ha-RR versus Sf-SS interaction; (**C**) Ha-SS interaction with Sf-RR and (**D**) Ha-RR interaction with Sf-RR.

In the interspecific combinations in soybean plants, there was a higher probability of survival (ρs) of susceptible *H. armigera* (ρs = 0.85) than of susceptible *S. frugiperda* (ρs = 0.55) (Ha-SS vs. Sf-SS interaction) (log rank test < 0.05; Figure 5A). For the other interactions, no significant differences were found between the curves of these two pest species (log rank test > 0.05) (Figure 5B–D).

The pairwise comparisons in different combinations between species in soybean plants also showed that the competitive performance of susceptible *H. armigera* (ρs = 0.85) in the interaction Ha-SS vs. Sf-SS was superior to the performance of *S. frugiperda* (ρs = 0.25) in the Ha-RR vs. Sf-RR interaction (log rank test < 0.05) and Ha-SS vs. Sf-RR interaction (log rank test < 0.05), although there was no difference in the survival of *H. armigera* when maintained in the combinations Ha-SS vs. Sf-SS and Ha-RR vs. Sf-SS (log rank test > 0.05). In the comparisons between the interspecific combinations within the species *S. frugiperda*, there was no significant difference in relation to the competitive performance of *S. frugiperda* between the analyzed competition combinations (log rank test > 0.05).

Survival in soybean did not differ between the phenotypic combinations evaluated within the intraspecific interactions of *S. frugiperda* (log rank test > 0.05). For *H. armigera*, the survival rate in the interaction between susceptible individuals (Ha-SS vs. Ha-SS interaction) (ρs = 0.725) was significantly higher (log rank test < 0.05) than in the interaction between resistant and susceptible strains (Ha-RR vs. Ha-SS interaction) (ρs = 0.475) and between resistant individuals (Ha-RR vs. Ha-RR interaction) (ρs = 0.55). Therefore, the lowest rate of cannibalism was observed in the interaction Ha-SS vs. Ha-SS.

In general, when comparing the mortality rate among insect species in soybean, that rate for susceptible *H. armigera* (Ha-SS vs. Ha-SS interaction) (1−ρs = 0.275) was significantly lower (log rank test < 0.05) than susceptible *S. frugiperda* (Sf-SS vs. Sf-SS interaction) (1−ρs = 0.80). Therefore, we observed a higher rate of mortality among susceptible *S. frugiperda* larvae than among susceptible *H. armigera* larvae. For the other phenotypic interactions, there was no significant difference (log rank test > 0.05).

### 3.2. Computational Model

#### 3.2.1. Intra- and Interspecific Interactions

**Figure 6:**
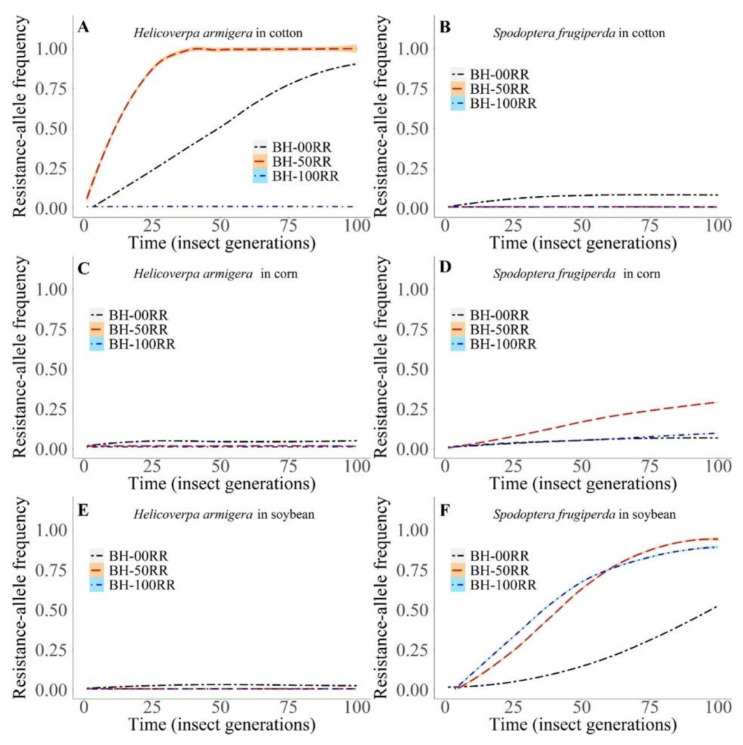
Absolute frequency of *Spodoptera frugiperda* and *Helicoverpa armigera* strains in intra- and interspecific interactions in cotton, corn, and soybean plants in the following scenarios of heterozygous behavior (BH): BH-00 RR, competition behavior of the heterozygote 100% equal to the susceptible ones; BH-50 RR, competition behavior of the intermediate heterozygote, i.e., 50% equal to the susceptible and 50% equal to the resistant individuals; BH-100RR, competition behavior of the heterozygote 100% equal to the resistant ones. (**A**) *H. armigera* in cotton. (**B**) *S. frugiperda* in cotton. (**C**) *H. armigera* in corn. (**D**) *S. frugiperda* in corn. (**E**) *H. armigera* in soybean. (**F**) *S. frugiperda* in soybean.

Considering the intra- and interspecific interactions in *H. armigera* in cotton, the unique case in which there was no evolution of resistance in this species in the simulated scenario of competition behavior of heterozygotes was 100% equal to resistant homozygotes (BH-100RR; Figure 6A). The evolution curves showed an asymptotic shape in the other cases (BH-00RR and BH-50RR), and in the intermediate competition behavior (50RR), growth was more intense. There was competitive exclusion of individuals with the susceptibility allele because the resistance allele reached the maximum frequency (Figure 6A).

With intra- and interspecific interactions in *S. frugiperda*, the unique case of evolution of resistance in cotton was when heterozygous individuals had 100% competition behavior equal to susceptible homozygotes (00 RR; Figure 6B). In this case, the curve reached stabilization over 30 generations of the insect and with a resistance allele frequency lower than 0.15 (Figure 6B).

In corn, there was no evolution of resistance in *H. armigera* populations (Figure 6C). In *S. frugiperda* in corn, the condition in which there was an increase in the resistance evolution curve was when the heterozygous strain showed intermediate competition behavior (50RR), reaching the highest frequency (0.47) over 38 insect generations (Figure 6D).

Similar to that observed in corn, there was no evolution of resistance in *H. armigera* populations in soybean (Figure 6E). In the case of *S. frugiperda* in soybean, there was an increase in the resistance evolution curves under the three analyzed conditions of heterozygous behavior (Figure 6F). The lowest rate of resistance evolution was found in the 00RR condition. In the other scenarios, 50 RR and 100 RR, the curves were sigmoidal and asymptotic, respectively (Figure 6F). Nevertheless, in these last two conditions, the proportion of the resistance allele approached the maximum frequency in the 100 RR scenario and reached this point in 50 RR (Figure 6F). Therefore, the partial (50 RR) or total (100 RR) similarity of the competition behavior of the heterozygotes with the resistant ones reflects an evident risk of extinction of insects carrying the *S. frugiperda* susceptibility allele when in intra- and interspecific interactions with *H. armigera* in soybean (Figure 6F).

#### 3.2.2. Intraspecific Interactions

**Figure 7:**
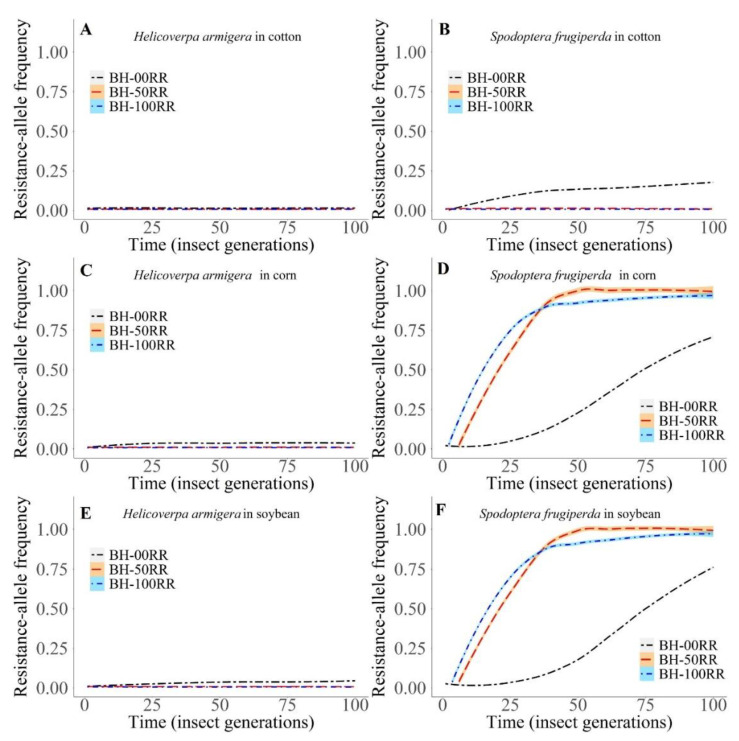
Absolute frequency of *Spodoptera frugiperda* and *Helicoverpa armigera* strains in intraspecific competition in cotton, corn, and soybean plants in the following scenarios: BH 00RR, competition behavior of the heterozygote 100% equal to the susceptible ones; BH 50RR, competition behavior of the intermediate heterozygote, i.e., 50%, equal to the susceptible and 50% equal to the resistant individuals, BH 100RR, competition behavior of the heterozygote, 100% equal to the resistant ones. (**A**) *H. armigera* in cotton. (**B**) *S. frugiperda* in cotton. (**C**) *H. armigera* in corn. (**D**) *S. frugiperda* in corn. (**E**) *H. armigera* in soybean. (**F**) *S. frugiperda* in soybean.

When we considered only the intraspecific interactions, there were no resistance evolution cases in *H. armigera* in all conditions and crops evaluated (Figure 7A,C,E). In the cotton simulations, the evolution of resistance in *S. frugiperda* with the behavior of the heterozygous 00 RR had an asymptotic response (Figure 7B) but reached an absolute frequency lower than 0.25 (Figure 7B). In corn and soybean, for *S. frugiperda*, the speed of resistance evolution was lower in the 50 RR and 100 RR conditions than in the 00 RR condition (Figure 7D,F); in addition, at one time the curves reached the maximum frequency (Figure 7D,F). Under the 50 RR and 100 RR behavioral similarity conditions in corn and soybean, there was a tendency toward competitive exclusion of insects carrying the susceptibility allele (Figure 7D,F).

## 4. Discussion

Our results support the hypothesis that behavioral competitivity pattern changes, measured by survival capacity and genotypic territoriality quantified by the frequency of occupation in the cellular automata, occurs according to the host plant/simulated agroecosystem. In corn plants, for example, resistant *S. frugiperda* were more competitive than susceptible *H. armigera*. In contrast, *H. armigera* showed better competitive performance than *S. frugiperda* when the competing species were a susceptible strain in soybean plants; the opposite result was observed in cotton plants. Therefore, our hypothesis that interactions involving competing species are influenced by the characteristics of the host plant was not contested because there is evidence that the host plant affects the competition of the species [19,20].

In general, computer simulations revealed that intra- and interspecific competition adversely affected the dynamics, measured by the relative frequency of *S. frugiperda* strains in cotton, corn, and soybean crops. In *H. armigera,* there was no effect of intraspecific interactions on the evolution of resistance in this insect, occurring only in the evolution of resistance in cotton. In corn, the competitive performance of susceptible *S. frugiperda* was superior to that of *H. armigera*. This would be expected by three factors: (1) because corn plants are the primary hosts of *S. frugiperda* [3]; (2) by the low relative fitness of *H. armigera* found naturally in corn plants [4], and naturally, a trade-off is found in the locomotion parameters of the diamide-resistant *H. armigera* strain, with an estimated reduction of 47% in the distance traveled and 36% in the movement speed in relation to the susceptible strain and the heterozygotes [21]; thus (3), the occurrence of a supposed fitness cost of resistant *H. armigera* larvae in corn in the absence of selective pressure could be expected.

In both corn and cotton plants, resistant *S. frugiperda* larvae did not allow the survival of susceptible *H. armigera* larvae. Although the interaction of resistant *S. frugiperda* individuals with *H. armigera* results in the death of susceptible *H. armigera* homozygotes in cotton, and considering that the rate of evolution of *S. frugiperda* was not high, this result did not significantly affect the propagation of the resistance allele in *H. armigera*. One of the factors that contributed to the low rate of resistance evolution of *S. frugiperda* in cotton is that resistant individuals have a low survival capacity when they compete with those susceptible to *H. armigera* in cotton plants, thus facilitating the population growth of *H. armigera* in that crop. In the 50 RR condition, the survival of *H. armigera* when in competition with susceptible homozygotes of the same species or of *S. frugiperda* was greater than in 00RR, accelerating the resistance evolution and even promoting the competitive exclusion of individuals carrying the susceptibility allele.

The competitive exclusion principle, or Gause’s principle, expresses that if two competing species coexist in a stable environment, then they do so because of the differentiation of their realized niches. If this differentiation does not occur or if it is obstructed by the habitat, then one of the competitors will eliminate or exclude the other, denying the other competitor its realized niche. The results of our study addressed a theme that extends the Gause principle to the genotypic level, as it revealed that there are cases in which the analyzed interactions can significantly influence the competitive exclusion of individuals with the susceptibility allele. Resistant individuals of *H. armigera* and *S. frugiperda* tend to not fit the niche for those heterozygous and homozygous susceptible individuals of *H. armigera* in cotton with intra + interspecific interactions and in *S. frugiperda* under conditions BH 50 RR and BH 100 RR with only intraspecific interactions.

In the case of *S. frugiperda*, the total similarity of the interspecific competitive performance of heterozygous individuals with the susceptible homozygote (00 RR) potentiated the survival of individuals carrying the resistance allele. In cotton, this was the unique condition in which there was evolution of resistance because, in the total similarity in relation to the susceptible strain, the heterozygous individuals presented superior competitive performance in relation to the 50 RR or 100 RR conditions, interfering with the population frequency of the resistance allele. However, the evolution of *H. armigera* resistance was much more pronounced than *S. frugiperda* in the 00 RR condition in cotton. In cotton plants, this result would also be expected for *H. armigera* to have a better competitive performance compared to *S. frugiperda*, with consequent evolution of more intense resistance. Antinutritional allelochemicals for *S. frugiperda* are found in cotton plants [3], such as gossypol, which promotes low initial survival in this host plant, despite the considerable damage caused by *S. frugiperda* in the crop in the absence of preferred hosts [22]. Conversely, *H. armigera* has a better relative fitness in cotton plants than in corn [4].

The highly competitive performance of the susceptible strain of *S. frugiperda* allowed the evolution of resistance of its species at lower rates under the BH 00 RR condition than under the BH 50 RR and BH 100 RR conditions in corn and soybean, with only intraspecific interactions and with intra- and interspecific interactions in soybean. The highly competitive performance of susceptible individuals of *H. armigera* in both intra- and interspecific interactions also disfavored the evolutionary process of resistance in soybean. This is clearly consistent with the hypothesis that the susceptible strain did not allow the survival of other strains to avoid interspecific competition, consequently increasing its fitness value in the presence of its phenotypic competitors.

Another factor that influenced the allelic frequency was the cannibalism rate. In our study, we demonstrated that cannibalism could vary beyond the species, as there may be an inter- and intragenotypic influence of the host plant species. The cannibalism in *S. frugiperda* and *H. armigera*, confirmed in our study and ascertained by other researchers [8], may in certain cases be advantageous, because feeding on individuals of the same species may represent a source of high-quality and readily accessible nutrients and restrict the vulnerability of the species to other competitors, predators, and food scarcity. Moreover, as observed in our study, it serves as a population factor to control the speed of resistance evolution, something that has not yet been documented. However, this effect may vary according to the insect species. Cannibal individuals of *H. armigera* have reduced size and impaired development [23]. *S. frugiperda* cannibal individuals had similar consumption rates, relative growth rates, and conversion efficiencies of ingested food compared to those fed only corn [24]. In our study in soybean, there was a low rate of cannibalism when there was competition between susceptible *H. armigera* larvae, contributing to the lack of resistance evolution. In corn, the highest rate of cannibalism was found in the interaction between resistant and susceptible individuals (interaction: Ha-RR vs. Ha-SS) of *H. armigera*. Similarly, cannibalism involving resistant and susceptible *S. frugiperda* larvae was more intense than in *H. armigera* when there was competition between susceptible (interaction: Ha-SS vs. Ha-SS) or resistant (interaction: Ha-RR vs. Ha-RR) in cotton. It is important to emphasize that interspecific interactions may also be influenced by temperature and pest ecology.

## 5. Conclusions

According to the experimental results and computer simulations, several conclusions were made: (i) there was evidence of behavioral changes in the pattern of competitiveness and phenotypic territoriality of *H. armigera* and *S. frugiperda* in cotton, corn, and soybean plants; (ii) susceptible *S. frugiperda* was more competitive than resistant *H. armigera* in cotton and corn plants; (iii) *H. armigera* was more competitive than *S. frugiperda* when both species were susceptible to insecticides in soybean and cotton plants; (iv) intra- and interspecific competition significantly affected the dynamics of *S. frugiperda* strains in cotton, corn, and soybean crops; (v) in the case of *H. armigera*, there was no effect of intraspecific interactions on the evolution of resistance of this insect, with the occurrence of evolution of resistance in cotton; and (vi) in terms of competitive exclusion, resistant individuals of *H. armigera* and *S. frugiperda* tended to not fit the niche for heterozygous and susceptible homozygotes of *H. armigera* in cotton with simultaneous intra- and interspecific interactions and in *S. frugiperda* in conditions of partial (BH 50 RR) and total similarity (BH 100 RR) of competitive behavior in relation to resistant individuals, with only intraspecific interactions in corn and soybean and with simultaneous intra- and interspecific interactions in soybean. The results obtained in this study are of great importance for understanding the impact of ecological factor competition on the evolution of insecticide resistance in lepidopteran pests.

## Data Availability

Not applicable.
